# Itaconic Anhydride as a Novel Bio‐Derived Solid Electrolyte Interphase Forming Additive for Lithium‐Ion Batteries

**DOI:** 10.1002/cssc.202501134

**Published:** 2025-06-30

**Authors:** Metin Orbay, Khai Shin Teoh, Massimo Melchiorre, Christof Neumann, Francesco Ruffo, Andrey Turchanin, Andrea Balducci, Juan Luis Gómez Urbano

**Affiliations:** ^1^ Institute for Technical Chemistry and Environmental Chemistry Friedrich‐Schiller University Jena Philosophenweg 7a 07743 Jena Germany; ^2^ Center for Energy and Environmental Chemistry Jena (CEEC Jena) Friedrich‐Schiller University Jena Philosophenweg 7a 07743 Jena Germany; ^3^ Dipartimento di Scienze Chimiche Università degli Studi di Napoli Federico II Complesso Universitario di Monte S Angelo, via Cintia 21 80126 Napoli Italy; ^4^ ISUSCHEM srl Piazza Carità, 32 80134 Napoli Italy; ^5^ Institute of Physical Chemistry Friedrich‐Schiller University Jena Lessingstraße 10 07743 Jena Germany

**Keywords:** additives, bio‐based, electrolytes, lithium‐ion batteries, solid electrolyte interphases, sustainability

## Abstract

In this work, itaconic anhydride (ITC) is introduced as a novel bio‐derived additive for lithium‐ion batteries. Its ability to create a stable solid electrolyte interphase (SEI) is evaluated in graphite electrodes and compared to vinylene carbonate (VC). The findings show that electrolytes consisting of 1 M lithium bis(trifluoromethanesulfonyl)imide in propylene carbonate and containing ITC and VC additives display similar physicochemical properties. The ability of ITC to form an effective SEI is demonstrated by reversible lithium intercalation during galvanostatic cycling and further corroborated by in situ Raman spectroscopy. Moreover, graphite and lithium iron phosphate (LFP) half‐cells display similar electrochemical performance in terms of rate capability and capacity retention along cycling for ITC‐ and VC‐based formulations. ITC undergoes a distinct reduction mechanism on graphite, forming a SEI layer containing C–O and COO^−^ species. Additionally, some insights into the plausible reaction pathways of the reduction byproducts associated with ITC are provided. In sum, this work aims to pave the way toward enhancing the overall sustainability of energy storage devices by exploring a novel bio‐based alternative to conventional petrochemical‐derived additives.

## Graphical abstract

This work presents itaconic anhydride as a novel bio‐derived additive able to form a suitable solid electrolyte interphase on graphite anodes and demonstrating similar performance to that of vinylene carbonate. Further, its decomposition mechanisms are investigated and a plausible pathway for solid electrolyte interphase formation is proposed.

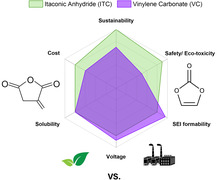

## 1 Introduction

With the ever‐expanding electrification in our modern life, ranging from electric vehicles to portable electronics and stationary storage systems, the demand for energy storage technologies has grown significantly. Lithium‐ion batteries (LIBs) have emerged as the dominant technology, widely recognized as the state‐of‐the‐art solution due to their high energy density reaching up to 280 Wh kg^−1^, along with superior efficiency, long cycle life, and cost‐effectiveness.^[^ ^1^ ^]^ As a result, LIBs have become indispensable across a broad range of industries. Forecasts indicate that by 2030, global battery production will increase substantially, with an anticipated total LIB cell capacity of 2.5 TWh compared to 0.5 TWh in 2020.^[^ ^2^ ^]^ However, this ongoing growth in battery production raises several concerns, especially from the resource supply perspective.^[^ ^3^ ^]^ The problems surrounding electrode raw materials are well‐known to the wider public and scientific community. In contrast, the same concerns surrounding the electrolyte components in LIBs are often overlooked. Currently, the state‐of‐the‐art formulation used in commercial LIBs typically consists of a mixture of carbonates, such as ethylene carbonate (EC) and dimethyl carbonate (DMC), with lithium hexafluorophosphate (LiPF_6_) as the primary salt.^[^ ^4^ ^]^ Furthermore, several compounds are included in the electrolyte formulation in small proportions as additives with the aim of fulfilling a required function (e.g., flame retardant, SEI formation).^[^ ^5^ ^]^ Some of these electrolyte components pose significant safety and environmental concerns. The LiPF_6_ salt not only requires the mining of several ores (i.e., phosphate rock, fluorite, lithium brines, or spodumene) for its production but also is known to decompose, releasing toxic and corrosive hydrogen fluoride, which significantly reduces the overall safety profile of LIB.^[^ ^4^ ^]^ On the other hand, the production of carbonate solvents and some of the conventional additives relies on the nonrenewable steam cracking of hydrocarbons, particularly naphtha, which is one of the largest contributors to greenhouse gas emissions within the petrochemical industry.^[^ ^6^ ^]^ In recent years, great efforts have been made in investigating greener solutions for the solvents (bio‐derived, petroleum byproducts) and salts (fluorine‐free).^[^ ^7^ ^]^ Nevertheless, there remains a significant gap in research concerning the implementation of eco‐friendly additives, particularly in the context of solid electrolyte interphase (SEI) stabilization.^[^ ^8^ ^]^ In this regard, despite the use of sustainable additives offers a promising pathway to reduce the environmental impact of the electrolyte, to the best of our knowledge, this area remains unexplored. To find a suitable SEI additive is anyway not an easy task since its selection is directed by several key parameters that would ensure both performance and stability in LIB systems.^[^ ^9^ ^]^ To support the formation of an SEI on the graphite anode, the additive needs to undergo reduction on the surface before the cointercalation of solvent molecules occurs (e.g., propylene carbonate). Decomposition products arising from the reduction of the additive should form a proper protective layer, acting as a barrier for continuous solvent reduction while being permeable to the desired cation (i.e., Li^+^). Additionally, the additive must display chemical stability with the salt, solvents, and other additives present in the electrolyte while maintaining compatibility with various anode and cathode chemistries. Given that the SEI is mostly composed of a polymerized layer and inorganic compounds, it is important for the selected additive to contain unsaturated bonds, which can promote the SEI formation through polymerization and reduction reactions.^[^ ^10^ ^]^ As previously mentioned, the ideal additive should be derived from bio‐based sources, offering a lower toxicity profile compared to traditional petrochemical‐derived additives like vinylene carbonate (VC), which is considered a chemical of high concern (BM–1).^[^ ^11^ ^]^ Moreover, while VC remains widely used for its effectiveness in SEI formation, its production, mainly derived from photochlorination of EC, involves energy‐intensive processes reliant on petrochemical raw materials such as natural gas or crude oil.^[^ ^12^ ^]^ Considering the aforementioned, we have identified itaconic anhydride (ITC) as an alternative candidate for this role. The terminal unsaturated bond and its terminal moiety make this compound a promising SEI‐active candidate. ITC is derived from itaconic acid, listed as one of the “Top 12 Platform Chemicals” that can be potentially produced from biomass fermentation, such as corn or beetroot.^[^ ^13^ ^]^ ITC can be produced directly from citric acid.^[^ ^14^ ^]^ These synthetic routes, represented in Figure S1, Supporting Information, highlight the precursor´s bio‐based origin and offer a pathway toward enhancing the sustainability of each component in the cell.^[^ ^15^ ^]^

Herein, we report about the use of ITC as a novel bio‐based SEI‐forming additive for LIBs. Initially, we investigated the physicochemical properties of an electrolyte containing 1 M lithium bis(trifluoromethanesulfonyl)imide (LiTFSI) in propylene carbonate (PC) with ITC and compared them with those of an identical electrolyte but containing VC. Next, we analyzed the electrochemical performance of this novel bio‐based additive in graphite and lithium iron phosphate (LFP) electrodes. Finally, a thorough analysis is carried out to understand the film‐forming ability of ITC on graphite electrodes and to propose plausible SEI formation pathways.

## 2 Results

### 2.1 Electrolyte Characterization

**Figure 1:**
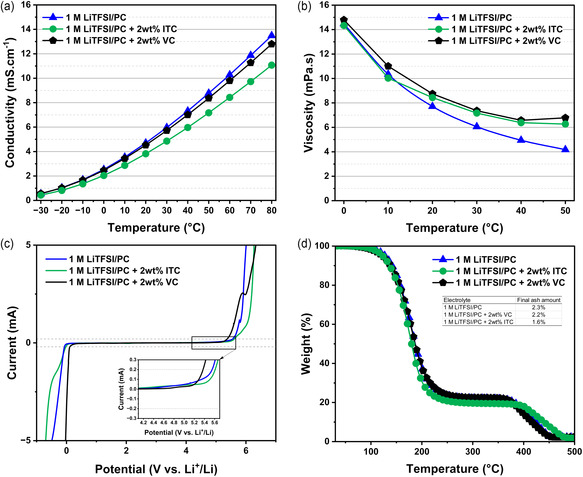
a) Conductivity values of noted electrolytes measured from −30 to 80 °C; b) viscosity values of corresponding formulations from 0 to 50 °C; c) electrochemical stability and d) dynamic TGA of labeled electrolytes including final ash amount.

In this study, we investigated the film‐forming ability of ITC by implementing it in an electrolyte composition known for its inability to form a suitable SEI layer on graphite: 1 M LiTFSI in PC.^[^ ^16^ ^]^ The physicochemical and electrochemical properties of 1 M LiTFSI in PC with ITC are compared to those of 1 M LiTFSI in PC with and without VC. The physicochemical properties of benchmark 1 M LiPF_6_ in EC:DMC (LP30) are also reported for the sake of comparison in the Supporting Information. **Figure** **1**a,b depicts the conductivity and viscosity values, respectively, measured for the aforementioned formulations in a wide range of temperatures. As expected, the benchmark LP30 electrolyte demonstrates the highest conductivity and lowest viscosity across all temperature ranges (Figure S2, Supporting Information). Pristine 1 M LiTFSI in PC shows slightly better transport properties than those measured for the formulations containing VC and ITC additives. Both additives lead to a small decrease in conductivity and an increase in viscosity, which agrees with previously reported literature.^[^ ^17^ ^]^ More in detail, at 20 °C, the 1 M LiTFSI in PC with 2 wt% VC electrolyte presents a conductivity of 4.53 mS cm^−1^ and a viscosity of 8.74 mPa·s, while slightly lower transport values are measured for 1 M LiTFSI in PC with 2 wt% ITC (3.83 mS cm^−1^ and 8.44 mPa·s). Regarding the electrochemical stability window of the electrolytes (Figure 1c), the additive‐free 1 M LiTFSI in PC electrolyte exhibits a similar oxidative limit when compared to the formulation featuring 2 wt% ITC (5.4 V vs. Li^+^/Li). In contrast, and in good agreement with previous reported studies, the incorporation of VC results in a slight decrease of the voltage window (5.2 V vs. Li^+^/Li).^[^ ^18^ ^]^ These findings indicate that the addition of ITC does not significantly compromise the electrochemical voltage window of the electrolyte. The thermal stability profiles registered for the VC and ITC‐containing electrolytes (Figure 1d) show an identical initial weight loss and a similar onset temperature for salt decomposition. Additionally, the ITC‐based formulation exhibited no evident reactivity when exposed to lithium metal for 10 days (Figure S3, Supporting Information), indicating good chemical stability against lithium metal. Overall, we can conclude that the physicochemical properties of VC and ITC in 1 M LiTFSI‐PC are similar. However, ITC stands out as a bio‐derived additive with enhanced safety that can promote the overall sustainability of the electrolyte. Relevant physicochemical and safety parameters summarized in Table S1 further highlight the potential of ITC as a viable and effective additive for the production of sustainable electrolytes.

### 2.2 Electrochemical and Structural Characterization

**Figure 2:**
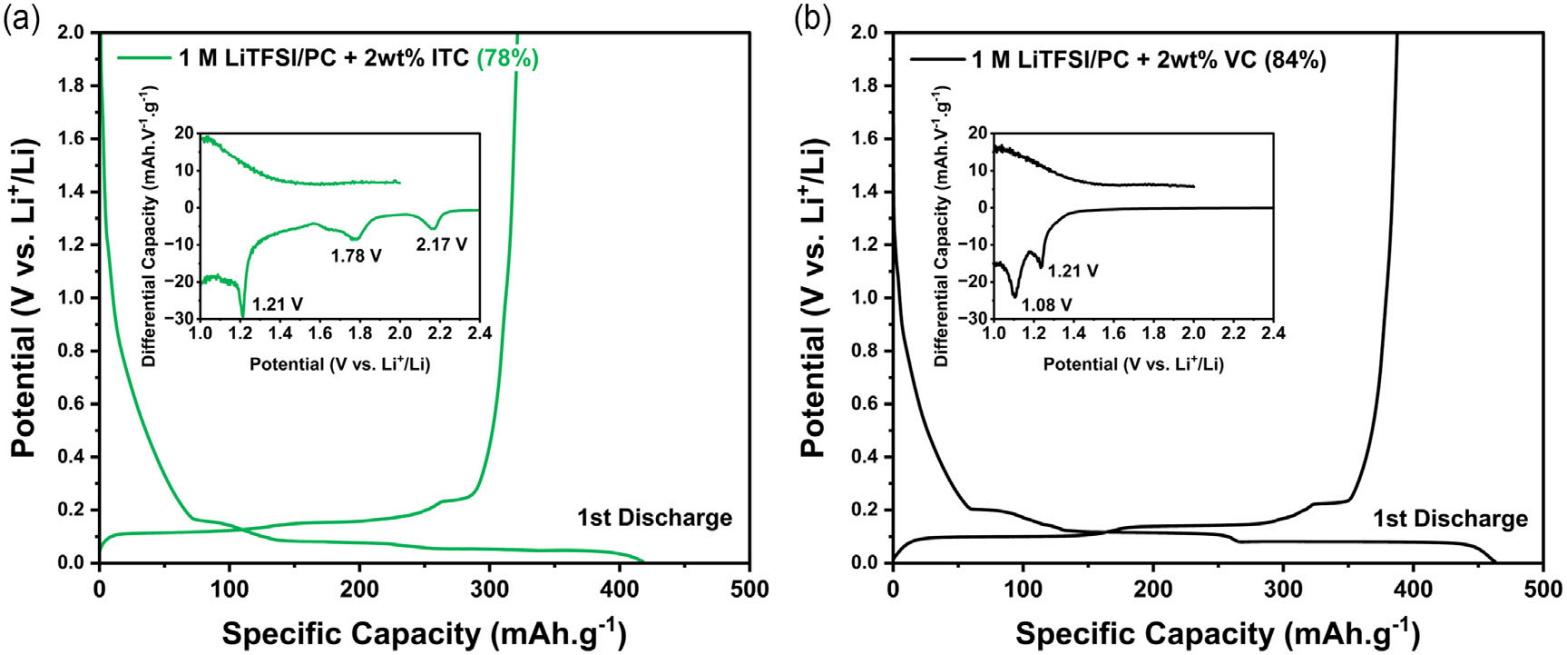
First charge–discharge profiles of graphite electrodes in 1 M LiTFSI in PC with a) 2 wt% ITC and b) 2 wt% VC at 0.05 C showing the first irreversible capacity for each additive. Inset: differential capacity of the first discharge showing three reduction currents at 1.21, 1.78, and 2.17 V versus Li^+^/Li in ITC and two reduction currents at 1.21 and 1.08 V versus Li^+^/Li in VC.

The suitability of ITC as SEI‐forming agent for LIB negative electrodes was investigated in a graphite half‐cell configuration. In addition, formulations of 1 M LiTFSI in PC with and without 2 wt% VC were evaluated for comparison purposes. As depicted in Figure S4, Supporting Information, the initial galvanostatic discharge of the cell using 1 M LiTFSI in PC reveals an infinite voltage plateau at ≈0.8 V versus Li^+^/Li. This well‐known phenomenon arises from the continuous cointercalation of PC‐solvated lithium ions within the graphene layers and the irreversible reductive decomposition of PC solvent.^[^ ^19^ ^]^ On the other hand, when ITC is added to the electrolyte, the discharge process is completed, and the three characteristic plateaus related to lithium staging into graphite are clearly visible (**Figure** **2**a). A similar behavior is observed for the VC‐based electrolyte represented in Figure 2b. These results suggest that ITC reduction is taking place before solvent intercalation occurs, creating a protective layer that prevents further electrolyte decomposition. Differential capacity curves plotted on Figure 2a inset for the ITC‐containing electrolyte reveal a complex reduction process, showing two broad peaks at 2.17 and 1.78 V versus Li^+^/Li, followed by a sharp peak at 1.21 V versus Li^+^/Li. This latter peak is similar to that observed for VC (Figure 2b inset). This extended SEI formation process led to lower initial coulombic efficiency values for ITC (78%) when compared to that of VC (84%). This phenomenon could be related to the presence of impurities in the ITC additive employed in this study or inherent side reactions. To gain further information, an electrolyte formulation consisting of 1 M LiTFSI and 2 wt% itaconic acid (ITC‐Ac) was also tested (Figure S4, Supporting Information). The use of ITC‐Ac as an additive did not provide SEI protection and led to graphite exfoliation, similarly to that measured for the pristine 1 M LiTFSI in PC electrolyte. Besides, a characteristic reduction process was observed at 1.78 V versus Li^+^/Li, which fits well with the peak observed for the ITC‐containing electrolyte. This result suggests that the presence of this molecule may play a role in the extended SEI formation mechanism observed for ITC.

**Figure 3:**
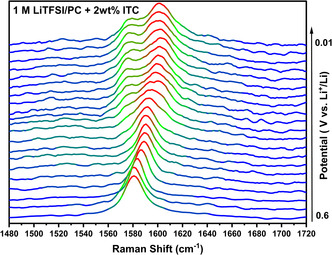
In situ Raman of 1 M LiTFSI in PC with 2 wt% ITC showing the shift and splitting of the G band at 1620 cm^−1^ upon lithiation, which indicates lithiation stages in graphite.

The lithium intercalation into graphite when employing the ITC‐based electrolyte was further investigated by in situ Raman spectroscopy (**Figure** **3**). The Raman spectra were continuously recorded across different applied potential ranges (full range spectra in Figure S5, Supporting Information). The splitting and shift of the graphite G band (around 1620 cm^−1^) upon lithiation further demonstrates the successful Li^+^ intercalation within the graphite structure, enabled by the SEI formed from the ITC decomposition products.^[^ ^20^ ^]^

**Figure 4:**
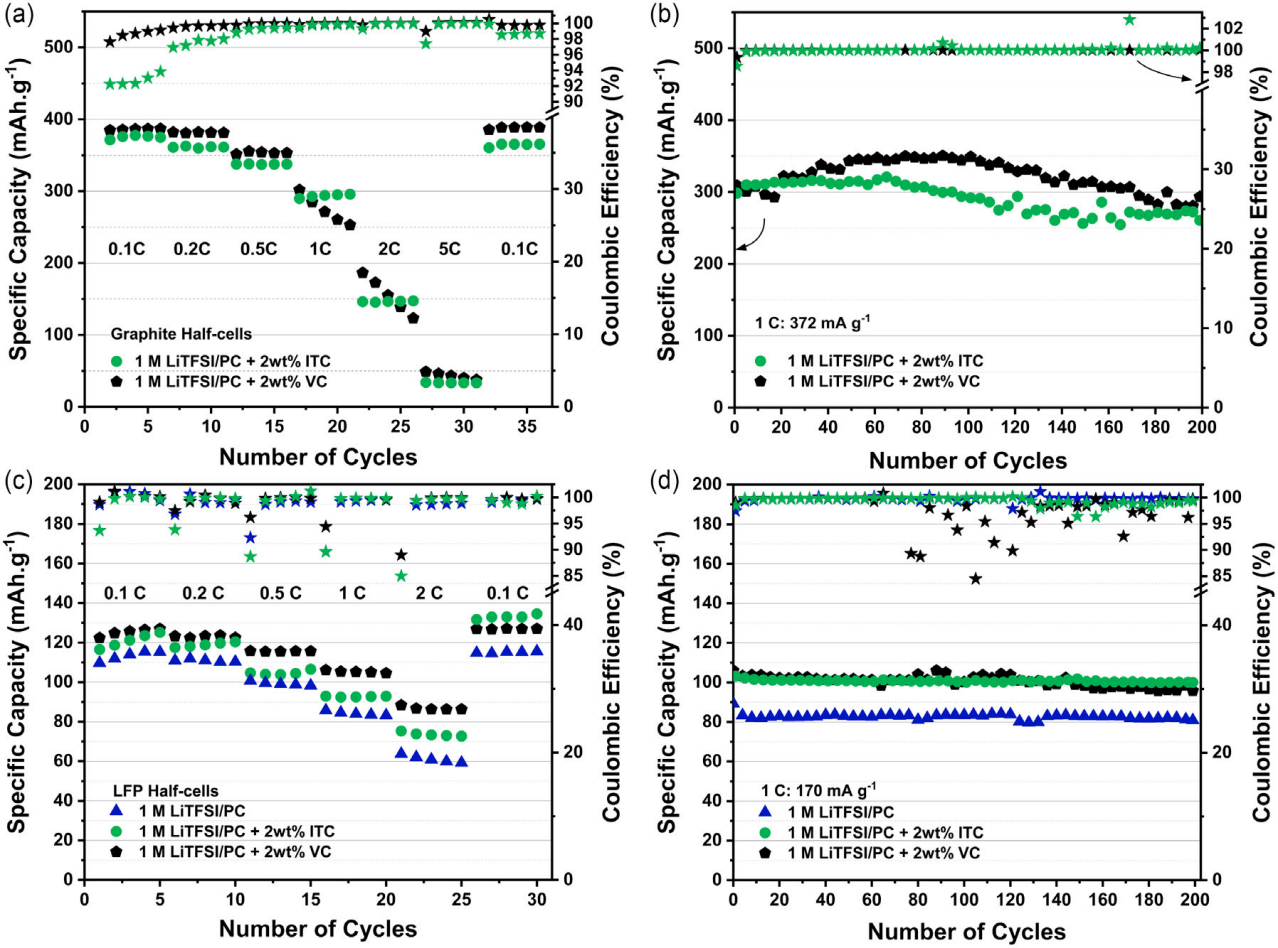
a) Rate capability tests over various C rates and b) cycling stability tests at 1 C for 1 M LiTFSI in PC with 2 wt% ITC and 2 wt% VC on graphite half‐cells, c) rate capability tests and d) cycling stability tests for the noted electrolytes on LFP half‐cells.

In order to evaluate the performance metrics of these electrolytes, electrochemical tests were performed. As shown in **Figure** **4**a, the rate performance of graphite electrodes combined with VC and ITC additives in 1 M LiTFSI in PC was assessed at various current densities. Both electrolytes demonstrated comparable capacities, with the ITC‐containing electrolyte delivering 372, 361, 337, 295, 146, and 33 mAh g^−1^ at 0.1, 0.2, 0.5, 1, 2, and 5 C, respectively. Similarly, the VC‐containing electrolyte achieved 385, 381, 352, 271, 146, and 43 mAh g^−1^ under the same conditions. However, ITC exhibits lower coulombic efficiency during the initial cycles at low current densities, which gradually increases with continued cycling. This behavior aligns well with the trend observed in the electrochemical impedance spectroscopy (EIS) measurements conducted during the rate capability test (Figure S6, Supporting Information). Charge transfer resistance (R_CT_) values calculated from the Nyquist plots are summarized in Table S2. At the initial stages of the test, the R_CT_ values of the ITC‐containing sample are one order of magnitude higher than those of its VC counterpart, indicating strong interfacial polarization.^[^ ^21^ ^]^ Nevertheless, both formulations display similar R_CT_ values by the end of the rate test, suggesting that the ITC‐based formulation requires a longer activation period. Exchange current density ($i^{0}$) values derived from R_CT_ are also summarized in Table S2 to facilitate comparison of the electrode/electrolyte kinetics. The long‐term cycling at 1 C performed after the rate capability test for the graphite half‐cells with the VC and ITC‐based formulations is illustrated in Figure 4b. The ITC‐based electrolyte demonstrated good cycling stability, achieving 91% capacity retention after 200 cycles, compared to 94% for the VC‐based electrolyte under identical conditions.

The 1 M LiTFSI in PC electrolytes, with and without the addition of ITC and VC, were further tested in LFP half‐cells to assess their performance with positive electrode materials. The rate capability test (Figure 4c) shows that the addition of either ITC or VC results in a slight enhancement in performance compared to the additive‐free electrolyte. More in detail, the ITC‐containing electrolyte achieves comparable capacity to its VC counterpart at low current densities, although slightly lower capacity values are measured at higher C rates. When the current density is restored to a lower value (0.1 C) at the end of the rate capability test, the capacity delivered by the ITC‐containing formulation surpasses that of the VC‐containing one. This behavior aligns well with the graphite half‐cell measurements, indicating a slow activation process for the ITC‐based electrolyte. The cycling test performed after the C‐rate test (Figure 4d) validated this finding, as both additive‐containing formulations deliver similar capacity values at 1 C, outperforming the additive‐free formulation. Overall, the ITC‐containing electrolyte exhibits good cycling stability at 1 C, with 97% capacity retention after 200 cycles. These results suggest that, despite a longer activation process required, the addition of ITC has a positive impact on the performance of LFP and graphite materials.

### 2.3 SEI Formation on Graphite

**Figure 5:**
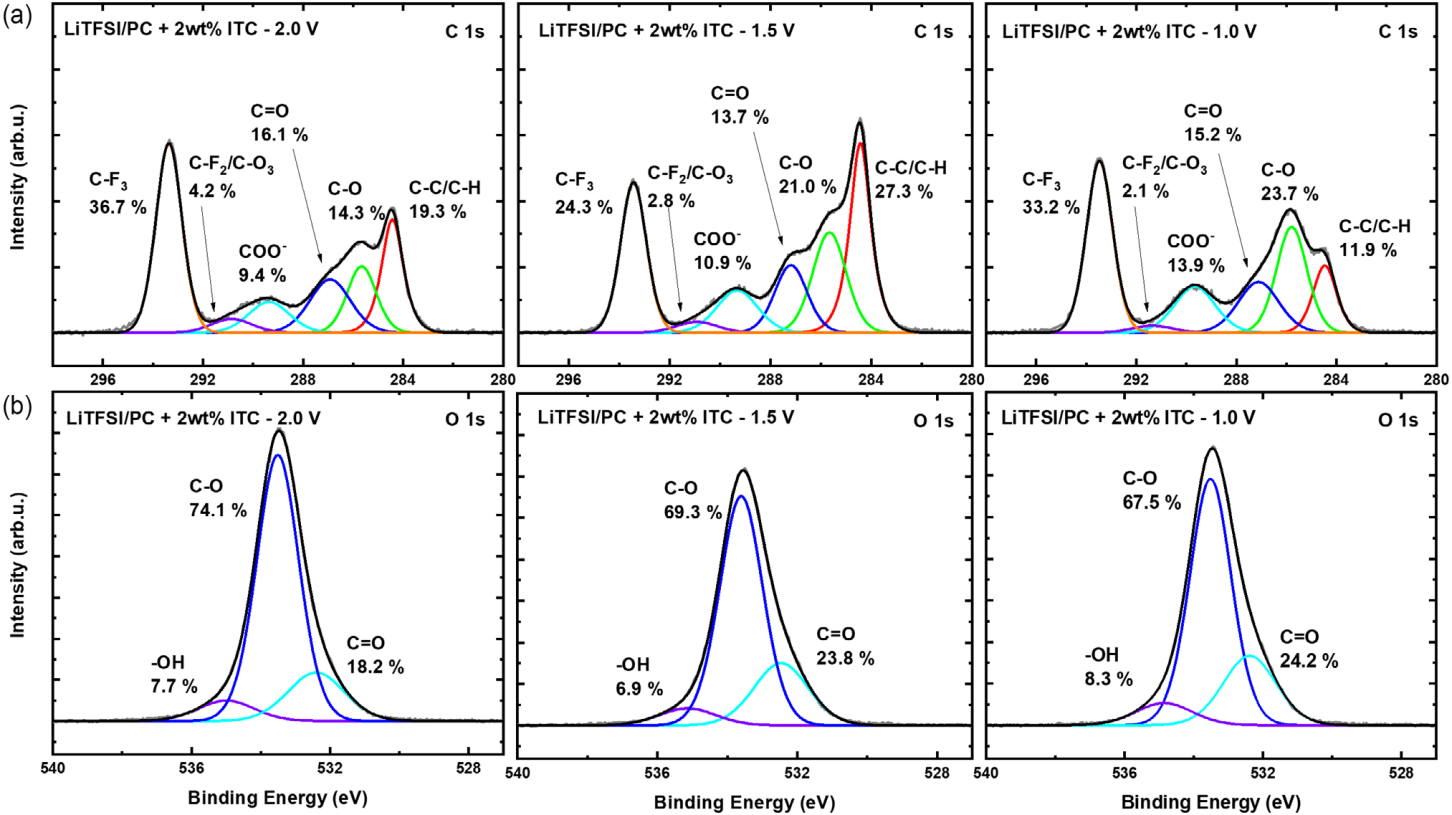
a) High‐resolution C 1*s* and b) O 1*s* XPS spectra for graphite electrodes in 1 M LiTFSI in PC with 2 wt% ITC measured after discharging to the cutoff potentials of 2.0, 1.5, and 1.0 V versus Li^+^/Li.

To gain a deeper understanding of the SEI‐forming ability of ITC, graphite half‐cells were discharged from open circuit potential to selected cut off potentials (2.0, 1.5, and 1.0 V vs. Li^+^/Li), and recovered graphite electrodes were analyzed via X‐ray photoelectron spectroscopy (XPS). As shown in **Figure** **5**, XPS analysis, particularly of the C 1*s* (carbon) and O 1*s* (oxygen) core peaks, revealed overall minimal differences among the electrodes at these potentials, which is expected given the high potentials where significant surface reduction products have yet to occur. However, some trends in species formation can be observed, specifically at the C 1*s* spectra where a clear increase in peak intensity at a binding energy (BE) of ≈285 eV, which corresponds to a C–O environment, and at a BE of 289.3 eV, indicative of a COO^−^ environment, was observed as the potential decreased. This suggests the gradual formation of C–O and COO^−^ species containing groups on the surface of the electrode. Moreover, the O 1*s* spectra displayed a C=O environment peak at a BE of ≈532 eV, which could be associated with the SEI arising from the electrolyte reduction byproducts such as alkyl carbonates, lithium carbonates, and other polyethylene oxide type compounds.^[^ ^22^ ^]^ The polymerization products can also be seen in the corresponding scanning electron microscopy (SEM) measurements carried out for the graphite electrodes (Figure S7, Supporting Information).

As a complementary study to the surface analysis, ITC‐ and VC‐based electrolytes were cycled between 0.005 and 2.0 V versus Li^+^/Li for two cycles in graphite half‐cells using an X‐type Swagelok cell. Following cycling, a detailed nuclear magnetic resonance (NMR) investigation was performed on the extracted bulk electrolytes and the corresponding cycled graphite electrodes, in order to study the SEI formation mechanisms in the presence of ITC and VC. As a reference, NMR measurements were also carried out for the freshly prepared electrolytes with ITC and VC. The ^1^H NMR analysis of the fresh electrolyte prepared with ITC revealed the presence of the citraconic anhydride (CIT) (≈0.2 mol%) and ITC‐Ac (≈0.1 mol%) as minor components, along with the main electrolyte components (ITC ≈ 1.8 mol%; PC ≈ 97.9 mol%). The presence of these impurities in the fresh electrolyte could be related to the complex SEI formation observed in Figure 2a. After the cycling process, the relative abundance of ITC in the bulk electrolyte decreases to 1.3 mol% and that of PC increases to 98.3 mol%, while the other components are nearly unaffected (CIT ≈ 0.3 mol%; ITC‐Ac ≈ 0.1 mol%). Comparatively, the relative abundance of VC remains constant at 2.3 mol% after cycling. Although the molar percentage decrease of ITC is small and VC results are almost constant, it is important to consider that the electrolyte volume in the X‐type Swagelok cells used is approximately ten times greater than in standard T‐type cells. Therefore, the diminution of SEI‐active compounds is expected to be more pronounced in regular cells. Spectra analysis details of bulk electrolytes are reported in Table S3. For the NMR analysis on the cycled graphite electrodes, they were extracted with acetonitrile‐d_3_ to investigate the side products retained on the graphite electrode surface after the galvanostatic cycling. In this case, the comparative analysis between ITC versus VC showed some similarities but also relevant differences (Figure S8, Supporting Information). Both samples showed a singlet at 3.04 ppm related to the presence of methanol, probably produced by some breakdown pathways. Moreover, for the sample extracted from the cycled electrode with ITC‐containing electrolyte, its signals are barely detectable, while the signals related to its impurities (CIT and ITC‐Ac) have higher intensities. Considering also the previous electrochemical analysis with ITC‐Ac (Figure S4, Supporting Information), this finding suggests that these two compounds are largely not involved in SEI formation. In the case of VC, its singlet at 7.30 ppm is not detectable. Besides these similarities, the two samples have different patterns in the range of 4.0–2.5 ppm. In the presence of VC, three multiplets are present and coupled with a doublet at 2.82 ppm (*J* = 4.4 Hz), a triplet at 2.76 ppm (*J* = 5.8 Hz), and a doublet at 1.04 ppm (*J* = 6.3 Hz). By the addition of D_2_O, the doublet and the triplet at 2.8–2.7 ppm almost disappear from the spectra, shifting to a lower field, which clearly indicates them as –OH protons. As confirmed by literature, the individuated compound is propylene glycol, most probably derived from PC hydrolysis (Figure S9, Supporting Information).^[^ ^23^ ^]^ Similar hydrolysis products have been found with electrolytes based on EC and DMC are used.^[^ ^24^ ^]^ This condition seems to be completely absent in the ITC‐derived sample, suggesting that the PC is not involved in the same breakdown processes as when VC is used. In the region between 4.0 ppm and 2.5 ppm, the only signal related to a different compound is a triplet‐like signal at 3.41 ppm coupled with signals at 2.16 ppm, hidden by the water signal but revealed by the correlation spectroscopy (COSY) analysis (Figure S10, Supporting Information). These signals may suggest the formation of 2‐(hydroxymethyl)succinic acid or analogs compounds (e.g., oligomers), which can be obtained by ITC anhydride‐opening and hydration.^[^ ^25^ ^]^ Therefore, based on the NMR spectral data, it allows us to suggest two possible reaction pathways for the SEI formation in the presence of ITC additive. One pathway could be driven by ITC's ring opening processes, resulting in linear structures (Figure S11a, Supporting Information), while the other may involve radical processes, leading to branched structures (Figure S11b, Supporting Information). It is noteworthy that ITC is a monomer known to undergo both radical polymerization and nucleophilic attack, especially in the presence of lithium ions, which could increase the electrophilic character of the carboxylic atoms and promote the ring opening reaction (Figure S12, Supporting Information).^[^ ^14^, ^26^ ^]^ For instance, water is a typical nucleophile able to open anhydrides, and despite its amount in the electrolyte being extremely low (<5–10 ppm), it could act as an initiator for the ring opening polymerization reaction. Moreover, besides water, some radical or negatively charged organic fragments typically derived from the electrolyte breakdown can act as nucleophiles and promote the anhydride‐opening process. Therefore, the presence of both oligomeric/polymeric structures can be assumed. However, as previously mentioned, the itaconic acid is not able to provide a stable SEI interface layer. Thus, in the case of the ITC, the pathway should begin with the anhydride reactivity, which later could be subjected to further processes (e.g., nucleophilic addition to the double bond, radical formation). The complex reactivity of ITC is in line with the reduced coulombic efficiency values observed during the first 5–10 cycles (Figure 4a), which could involve different mechanisms and kinetics. Moreover, the ability of cyclic anhydrides to provide protective layers in energy storage has already been reported (e.g., succinic and glutaric anhydrides), and ITC might act similarly.^[^ ^27^ ^]^ While these insights provide a compelling picture, future and dedicated studies will be conducted to elucidate the SEI formation mechanism driven by ITC.

### 2.4 Postcycling Studies on Graphite Electrodes

**Figure 6:**
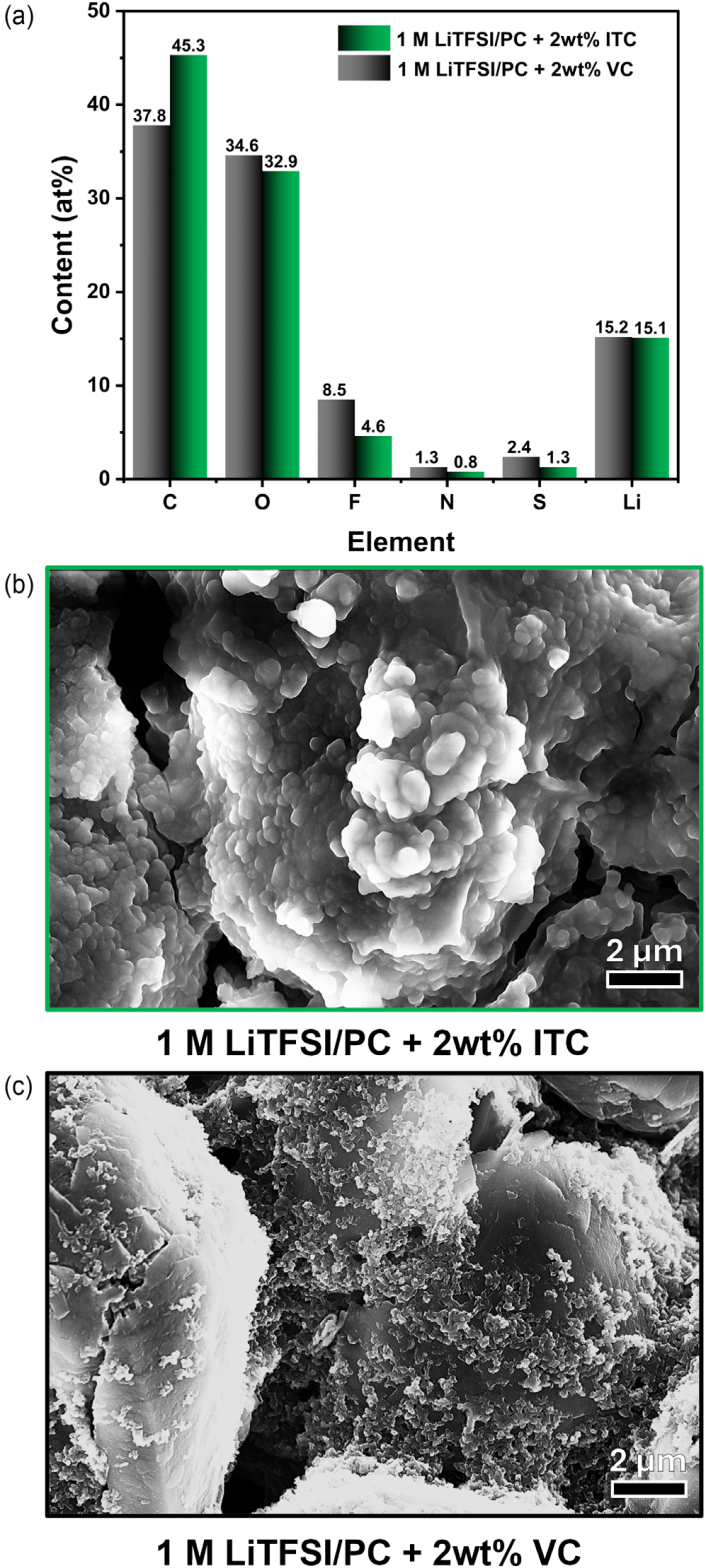
a) Atomic content (at%) of each element present in the SEI for VC‐ and ITC‐containing electrolytes after cycling. b) SEM images of the graphite electrodes cycled in 1 M LiTFSI in PC with 2 wt% ITC and c) 2 wt% VC.

To gain further insights into the influence of cycling on the SEI composition, XPS and SEM analyses were conducted on the electrodes after long‐term cycling. As shown in **Figure** **6**a, all electrodes present O, Li, F, S, and N after cycling, although their relative content differs for ITC and VC. Cycling in VC‐based electrolyte resulted in higher relative contents of F, N, S, and O. On the other hand, the C content was higher for the ITC formulation. These observations suggest a higher relative amount of organic species for the layer created after cycling with the ITC additive. Nevertheless, it is worth pointing out the formation of analogous species, such as Li_2_CO_3_, observed at a BE of ≈55.7 eV. On the other hand, both samples display signs in the F 1*s* spectra that can be ascribed to LiF (BE≈685.4 eV) and CF‐containing species. It is worth remarking that a higher relative amount of LiF species is observed for the ITC sample, which could indicate a higher decomposition of the LiTFSI salt during cycling (Figure S13, Supporting Information).^[^ ^28^ ^]^ The SEI morphology was investigated using SEM imaging (Figure 6b,c). The presence of a visible polymer matrix for both electrolytes related to the decomposition products can be clearly observed. Nevertheless, differences in morphology can be observed, with the ITC‐based electrolyte producing a thicker SEI layer. Although the precise composition and thickness of this layer are challenging to establish, it likely results from further polymerization of the ITC‐containing electrolyte to create a passivating layer. This evidence is supported by observations from the first cycle coulombic efficiency, polarization in EIS measurements, and XPS analysis.

## 3 Conclusion

This study introduces ITC as a novel bio‐derived electrolyte additive. Detailed physicochemical and electrochemical analyses reveal that electrolytes containing ITC and VC demonstrate similar physicochemical properties, and that ITC also presents the ability to form an effective SEI on the surface of graphite. The formulations containing ITC demonstrated suitable rate capability and cycling stability in graphite and LFP half‐cells. More in detail, the 1 M LiTFSI in PC + 2 wt% ITC electrolyte was able to retain 91% of its initial capacity after 200 charge/discharge cycles at 1 C (94% for VC) in graphite half‐cells. However, it is worth mentioning that ITC requires a prolonged activation process, which contributes to increased impedance and lower initial coulombic efficiency values. Ex situ XPS measurements performed to understand the step formation of the SEI layer revealed a gradual increase in C–O and COO^−^ species containing groups up to 1.0 V versus Li^+^/Li cut off potential due to ITC reduction upon first discharge. According to the used additive, NMR investigation provided different insights into the bulk electrolyte composition before and after cycling, as well as into the molecular structures retained on the graphite surface. Additionally, the XPS analysis of cycled graphite electrodes indicated a similar elemental content in the SEI composition for VC and ITC‐based formulations. However, the larger content of C species and relatively lower amount of F, N, and S, suggested the presence of a more organic passivation layer for the ITC formulation.

## 4 Experimental Seciton

4.1

4.1.1

##### Electrolyte Preparation and Characterization

PC (Sigma Aldrich, <99.7%, anhydrous), LiTFSI (99.9%), and commercial LP30 (99.9%) were purchased from Solvionic. ITC (ThermoFisher Scientific, 98%), itaconic acid (Sigma Aldrich, <99.9%), and VC (ThermoFisher Scientific, 98%) were used without prior purification and stored below 8 °C. The additives were added as wt% (%wt.) of the total electrolyte.

Conductivity measurements were performed using a ModulabXM ECS potentiostat. The cell was placed in a climatic chamber for temperature control. Conductance was determined using alternating current resistance of the electrolyte placed between parallel platinum electrodes with a known cell constant and measured in temperatures ranging from −30 to 80 °C.

Viscosity measurements were carried out using an AntonPaar MCR 102 rheometer equipped with a CP50‐0.5 cone‐plate system by sampling 400 μl of electrolyte with a constant shear rate of 1000 s^−1^ in a temperature range from 0 to 50 °C. The plate temperature during sample application was 0 °C.

Thermogravimetric analysis (TGA) measurements were carried out using a PerkinElmer STA 6000. Dynamic TGA measurements were performed using a 10 °C.min^−1^ ramp and nitrogen as the carrier gas.

##### Electrode Preparation

Graphite‐based inks were prepared by mixing 90% wt. active material (C‐Nergy Actilion GHDR 15‐4), 5% wt. of conducting agent (Super C65 from Imerys), and 5% wt. of binder (sodium carboxymethyl cellulose CMC2000 from Walocel) in water. The mixture was subjected to impact milling employing a high impact ball mill (Pulverisette 23, Fritsch, 10 min at 50 Hz). For LFP electrode preparation, the active material LiFePO_4_ (Südchemie) was mixed with carbon black as conducting agent (Super C65 from Imerys) and a binder solution in the mass ratio 90:5:5. The binder solution consists of 3.75 wt% CMC2000 (Walocel) and 1.25 wt% styrene‐butadiene rubber (SBR, Nanografi) in distilled water. Subsequently, graphite mixture was cast onto copper foil, while the LFP slurry was coated onto etched aluminum foils. The aluminum foils were etched in a 5% w/v KOH solution bath at 60 °C for 1 min. The electrode films were dried overnight under vacuum at 60 °C. Electrodes with an area of 1.13 cm^2^ were punched out from the cast and dried under vacuum at 60 °C for 12 h before use. The mass loadings of graphite and LFP electrodes were 1–2 mg.cm^−2^ and 2.7–3.5 mg.cm^−2^, respectively.

##### Electrochemical Characterization

The electrochemical stability window of the electrolytes was determined using linear sweep voltammetry (scan rate 1 mV.s^−1^) employing a platinum disc as working electrode and an oversized activated carbon as counter electrode. Polished silver wire was used as a quasireference electrode (current threshold of ± 0.1 mA). The electrochemical performance of the electrolyte additive against graphite and LFP electrodes was investigated utilizing Swagelok cells in a half‐cell configuration. Graphite and LFP electrodes were used, respectively, as the working electrode versus a lithium metal disc, serving simultaneously as counter and reference electrode. For graphite half‐cells, the current densities applied were calculated according to the theoretical capacity of graphite (1 C: 372 mA.g^−1^). Graphite electrodes were cycled from 0.005 to 2.0 V versus Li^+^/Li. EIS measurements were conducted in the fully charged state (2.0 V vs. Li^+^/Li) after one hour of rest over frequency ranges from 100 kHz to 100 mHz, and the extracted data were fitted using Zview software. Exchange current density ($i^{0}$) values were calculated according to

$$i^{0}=\frac{RT}{nFR_{CT}A}$$

where R is the gas constant, T is the absolute temperature, n is the number of charges transferred, F is the Faraday's constant, R_CT_ refers to the charge transfer resistance, and A to the electrode area (1.131 cm^2^). Galvanostatic measurements were performed on LFP half‐cells within a potential range of 3.0–3.8 V versus Li^+^/Li, with 1 C corresponding to 170 mA.g^−1^. All tests were carried out using Biologic VMP‐3 and Arbin LBT21084 potentiostats. Cell assembly and electrolyte preparation were performed in an argon‐filled glovebox (O_2_ and H_2_O < 1 ppm).

##### In Situ and Postmortem Characterization

In situ Raman measurement was carried out using a Renishaw InVia Raman microscope (532 nm, 2.5 mW) coupled with Biologic SP‐150 potentiostat in EL‐CELL ecc‐Opto‐Std cell configuration. For postmortem analysis, graphite electrodes were extracted under an argon atmosphere at the specified cutoff potentials and rinsed with PC. The excess of solvent was removed using an absorbent tissue, and the electrodes were left to dry. XPS was measured using a K‐Alpha X‐ray Photoelectron Spectrometer System (Thermo Fisher Scientific) with a monochromatic X‐ray source (Al K_α_) with a spot diameter of 400 μm and an electron detector with 0.5 eV energy resolution. A flood gun was employed for charge compensation. The spectra were calibrated using the C 1*s* peak (284.6 eV) and fitted using Voigt functions after background subtraction. SEM images were taken with a Zeiss Sigma VP at a beam energy of 15 kV and the use of the in‐lens detector of the system. For NMR investigation, graphite half‐cells were assembled with the studied electrolytes using X‐type Swagelok cells.^[^ ^29^ ^]^ In each cell, 1.5 mL of electrolyte was used to ensure a sufficient electrolyte reservoir for analysis. Two galvanostatic charge/discharge cycles within the potential range of 0.005–2.0 V versus Li^+^/Li were applied to the graphite half‐cells. After that, the cycled electrolytes and graphite electrodes were extracted respectively in the argon‐filled glovebox. NMR spectroscopy analysis, involving ^1^H and COSY, was performed using a Bruker Avance Ultrashield 400 MHz apparatus (Bruker Corporation) using CD_3_CN (Sigma Aldrich, 99.8 atom% D, contains 0.03 vol% TMS) and D_2_O (Sigma Aldrich, 99.9 atom% D) as solvents.

## Conflict of Interest

The authors declare no conflict of interest.

## Supporting information

Supplementary Material

## Data Availability

The data that support the findings of this study are available from the corresponding author upon reasonable request.
