# Supplementary material for: Itaconic Anhydride as a Novel Bio‐Derived Solid Electrolyte Interphase Forming Additive for Lithium‐Ion Batteries
Source: ChemSusChem. 2025 Jun 30;18(16):e202501134. doi: 10.1002/cssc.202501134 (PMC12330324; doi:10.1002/cssc.202501134)
Supplement: Supplementary file 1 — Supplementary Material [file CSSC-18-e202501134-s001.pdf]

Supporting Information

Itaconic Anhydride as a Novel Bio-derived Solid Electrolyte Interphase Forming Additive for Lithium-Ion Batteries

Metin Orbay[a,b], Khai Shin Teoh[a,b], Massimo Melchiorre[c,d], Christof Neumann[b,e], Francesco Ruffo[c], Andrey Turchanin[b,e], Andrea Balducci*[a,b], Juan Luis Gómez Urbano*[a,b]

[a] Institute for Technical Chemistry and Environmental Chemistry, Friedrich-Schiller University Jena. Philosophenweg 7a, 07743 Jena, Germany.

[b] Center for Energy and Environmental Chemistry Jena (CEEC Jena). Friedrich-Schiller University Jena. Philosophenweg 7a, 07743 Jena, Germany.

[c] Dipartimento di Scienze Chimiche, Università degli Studi di Napoli Federico II, Complesso Universitario di Monte S. Angelo, via Cintia 21, 80126, Napoli, Italy

[d] ISUSCHEM srl, Piazza Carità, 32, 80134, Napoli, Italy

[e] Institute of Physical Chemistry, Friedrich Schiller-University Jena, Lessingstraße 10, 07743 Jena, Germany

*E-mail: juanlu.gomez.urbano@uni-jena.de and andrea.balducci@uni-jena.de

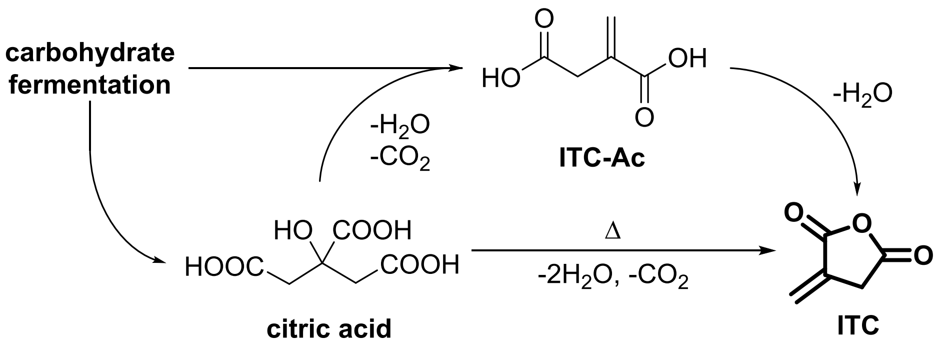

Figure S1. Synthetic routes to itaconic anhydride (ITC) by itaconic acid (ITC-Ac) dehydration and citric acid thermic treatment (decarbonylation/dehydration).

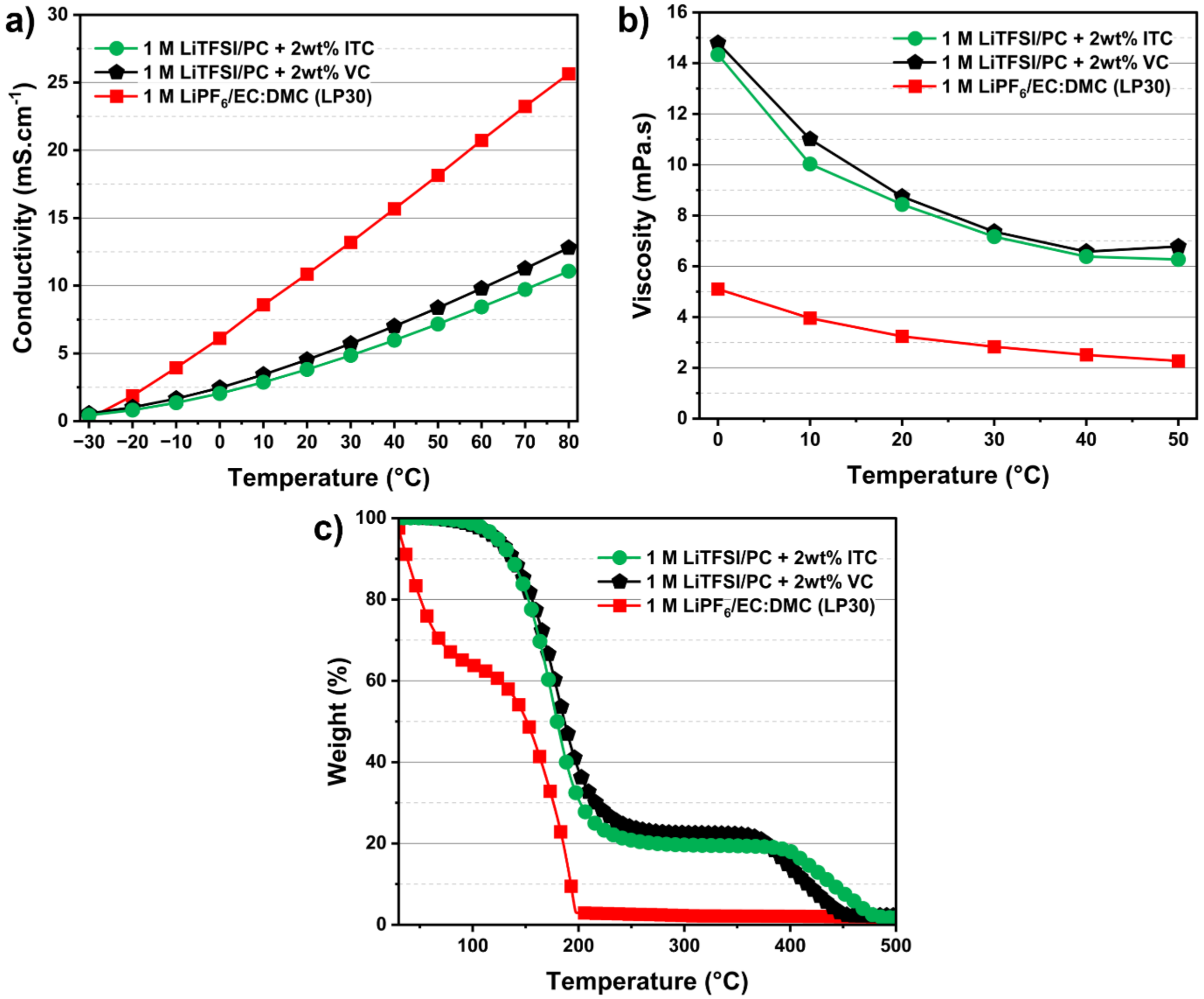

Figure S2. (a) Conductivity values of noted electrolytes measured from -30 to 80 °C; (b) viscosity values of corresponding formulations from 0 to 50 °C and (d) dynamic thermogravimetric analysis of labelled electrolytes.

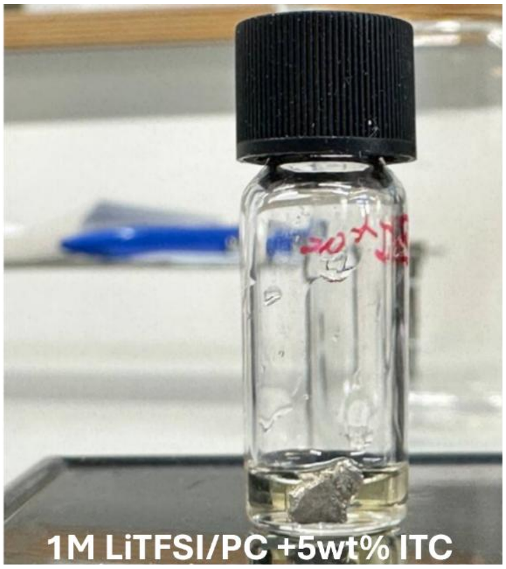

Figure S3. Itaconic anhydride (ITC) containing electrolyte after 10 days in contact with Li metal. No reactivity is visually observed (viscosity or color change).

Table S1. Physicochemical properties and other relevant parameters of pristine ITC and vinylene carbonate (VC) and associated formulations employing lithium bis(trifluoromethanesulfonyl)imide (LiTFSI) as salt and propylene carbonate (PC) as solvent. If a reference is not placed, the measurement was determined experimentally.

PARAMETER	ITACONIC ANHYDRIDE (ITC)	VINYLENE CARBONATE (VC)
**Conductivity in 1 M LiTFSI in PC (20 °C)**	3.83 $mS.cm^{-1}$	4.53 $mS.cm^{-1}$
**Viscosity in 1 M LiTFSI in PC (20 °C)**	8.44 mPa.s	8.74 mPa.s
**Boiling Point**	115 °C [1]	162 °C [2]
**Melting Point**	70 °C [1]	22 °C [2]
**Cost**	64.71 €.25 g^{-1} [1]	405 €.25 g^{-1} [2]
**Anodic Limit Measured In 1 M LiTFSI in PC**	5.4 V vs Li^+/Li	5.2 V vs Li^+/Li
**Eco-Toxicity**	LOW (classified as irritant) [3]	HIGH (Toxic, health hazard, environmental hazard, corrosive) [4]

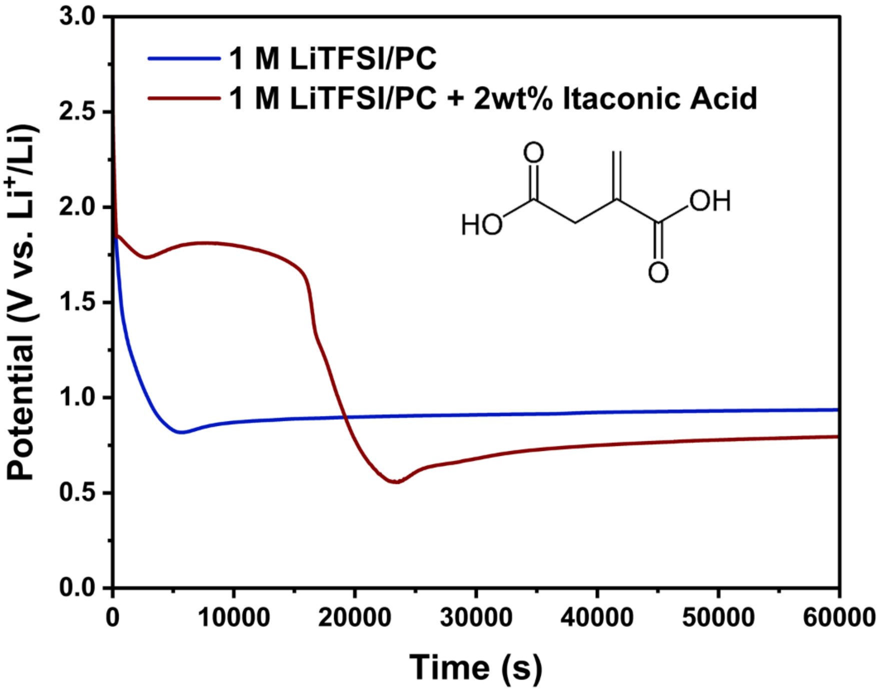

Figure S4. First cycle discharge of 1 M LiTFSI in PC without and with itaconic acid. The plateau at around 0.8 V vs. Li^+/Li indicates the precursor to itaconic anhydride does not provide any protection or SEI formation to graphite anodes.

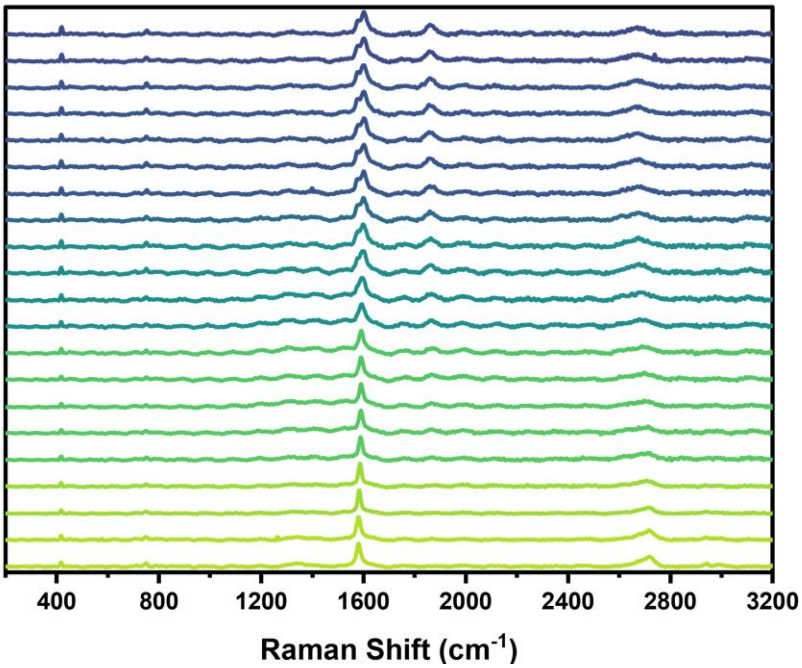

Figure S5. Expanded view from 3200 to 200 cm^{-1} shift of graphite electrodes measured employing ITC-containing electrolyte during lithiation. Splitting of G band around 1620 cm^{-1} shift can be observed. The appearance of peak at 1850 cm^{-1} which is interpreted as lithium carbide [5] indicating a possible onset of lithium plating due the in situ Raman cell configuration overpotentials.

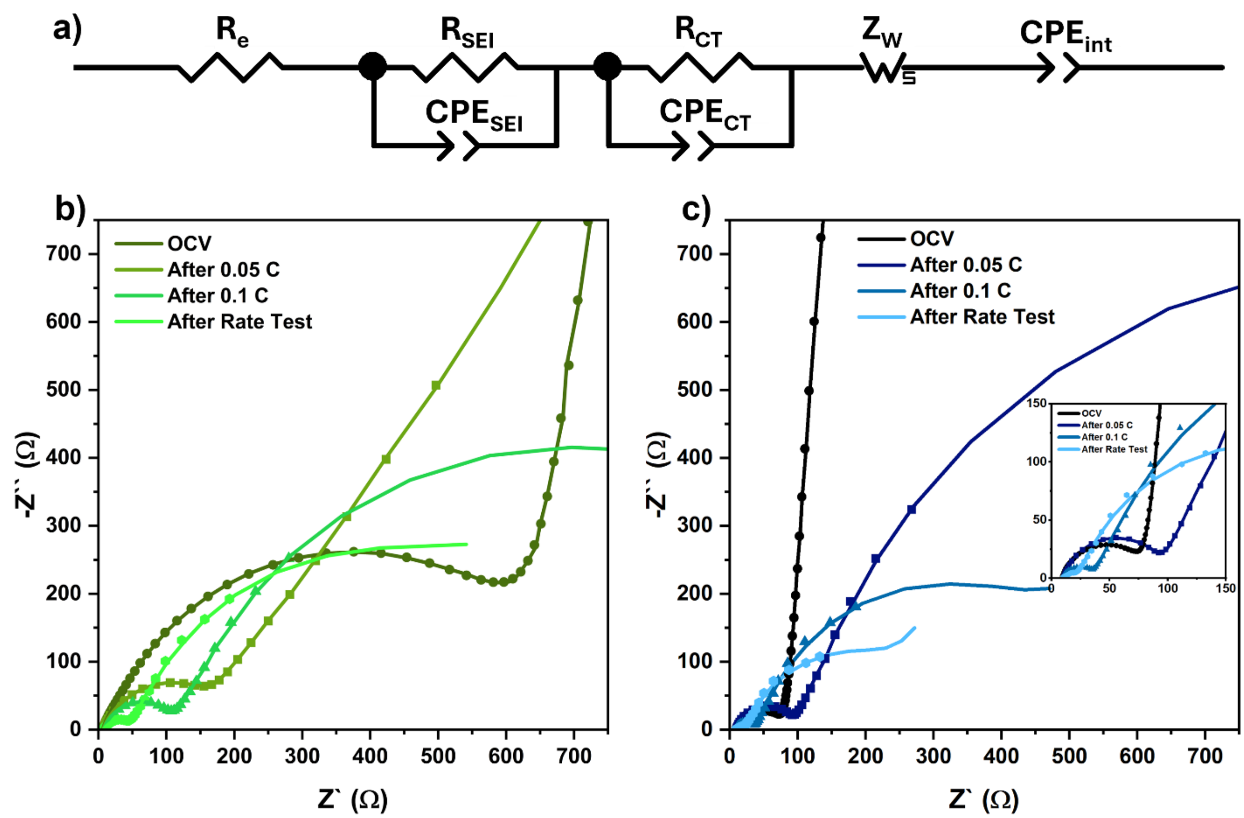

Figure S6. a) Equivalent circuit, and b) Nyquist plots of ITC and c) VC-containing electrolytes measured at noted cycling parameters. Solid lines correspond to experimental result and symbols to fitted data using the equivalent circuit.

Table S2. Resistance values, exchange current density values, and goodness of the fitting calculated for ITC and VC-containing electrolytes corresponding to measurements in Figure S6.

Sample		R_e (Ohm)	R_{SEI} (Ohm)	R_{CT} (Ohm)	i_0 ($mA.cm^{-2}$)	Chi^2
1 M LiTFSI in PC + 2wt% ITC	**OCV**	6.23	14.01	609.9	0.0366	5.37E-6
	**After 0.05 C**	6.82	34.12	101.2	0.2205	3.24E-6
	**After 0.1 C**	7.61	36.72	56.75	0.3934	2.53E-5
	**After Crate**	7.14	14.27	20.34	1.0975	1.24E-4
1 M LiTFSI in PC + 2wt% VC	**OCV**	9.39	5.80	59.57	0.3747	2.14E-6
	**After 0.05 C**	10.56	25.51	53.68	0.4159	2.19E-5
	**After 0.1 C**	9.60	6.12	17.30	1.2903	1.77E-4
	**After Crate**	9.63	3.82	5.66	3.9440	3.48E-5

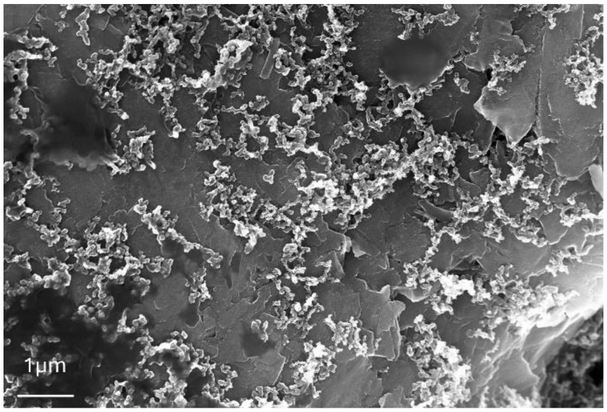

1 M LiTFSI/PC + 2wt% ITC (2.0 V cutoff)

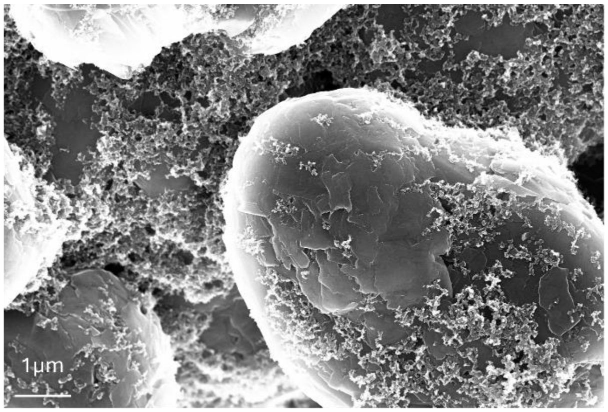

1 M LiTFSI/PC + 2wt% ITC (1.5 V cutoff)

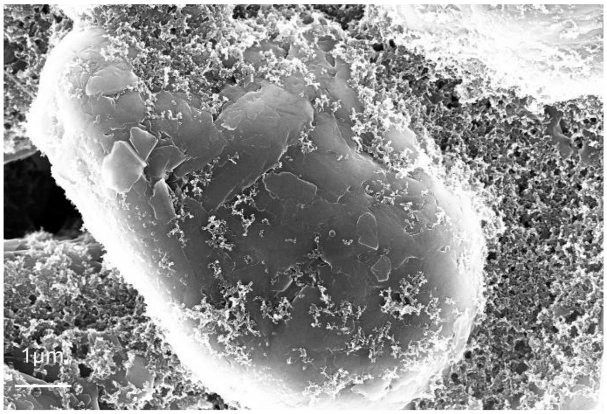

1 M LiTFSI/PC + 2wt% ITC (1.0 V cutoff)

Figure S7. SEM imaging of 1 M LiTFSI in PC +2 wt% ITC at different cutoff potentials. More polymerization products are seen as the voltage cutoff decreases.

Table S3. ***^{1}H NMR data analysis for fresh and aged PC/LiTFSI electrolytes with ITC and VC as additives. Relative composition excludes LiTFSI and other minor impurities.***

COMPOUNDS	*Abbreviations*	*Signals [ppm]*	*Integrals [a.u.]*		*Relative composition [mol%]*	
PC/ITC electrolyte			*Fresh*	*Aged*	*Fresh*	*Aged*
Itaconic anhydride	*ITC*	*6.39 (1H)*	*1.80*	*1.30*	*1.8*	*1.3*
Citraconic anhydride	*CIT*	*6.75 (1H)*	*0.23*	*0.30*	*0.2*	*0.3*
Itaconic acid	*ITC-Ac*	*6.21 (1H)*	*0.10*	*0.10*	*0.1*	*0.1*
Propylene carbonate	*PC*	*4.85 (1H)*	*100.00*	*100.00*	*97.9*	*98.3*
PC/VC electrolyte						
Vinylene carbonate	*VC*	*7.30 (2H)*	*4.80*	*4.73*	*2.3*	*2.3*
Propylene carbonate	*PC*	*4.85 (1H)*	*100.00*	*100.00*	*97.7*	*97.7*

Complete signals list for ITC additive

Itaconic anhydride: ^{1}***H NMR 400 MHz δ, 6.39 (t, J=2.8 Hz, 1H), 5.89 (t, J=2.4 Hz, 1H), 3.60 (t, J=2.6 Hz, 2H);***

Citraconic anhydride: ^{1}***H NMR 400 MHz δ, 6.75 (q, J=1.7 Hz, 1H), 2.11 (d, J=1.7 Hz, 3H);***

Itaconic acid: ^{1}***H NMR 400 MHz δ, 6.21 (s, 1H), 5.73 (s, 1H), 3.27 (s, 2H);***

Vinylene carbonate: ^{1}***H NMR 400 MHz δ, 7.30 (s, 2H).***

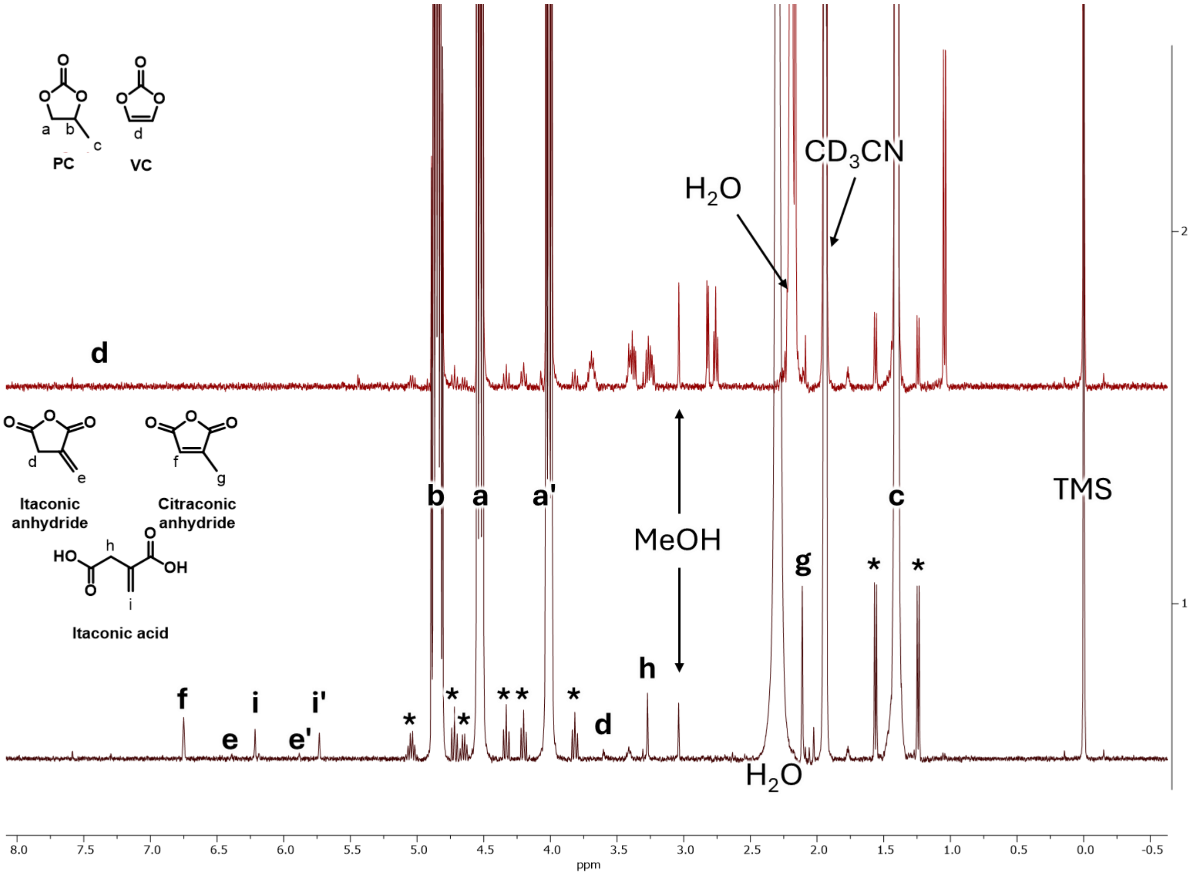

*Figure S8. ^{1}H NMR spectra of graphite electrodes extracted with CD_3CN previously cycled with PC/LiTFSI-VC (top) and PC/LiTFSI-ITC (bottom) electrolytes. Signals labelled with * refer to ^{13}C satellites.[6]*

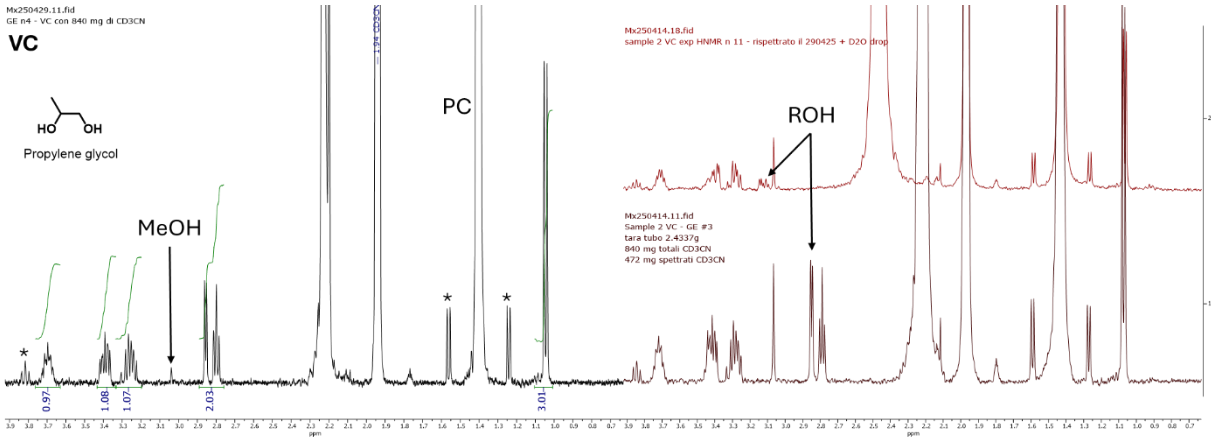

*Figure S9. Relevant portion of ^{1}H NMR investigation of graphite electrode cycled with PC/LiTFSI-VC electrolyte and extracted in CD_3CN. On the left are reported the details related to the presence of propylene glycol. On the right is reported the spectra comparison before (down) and after (top) the addition of D_2O. Signals labelled with * refer to ^{13}C satellites.[7]*

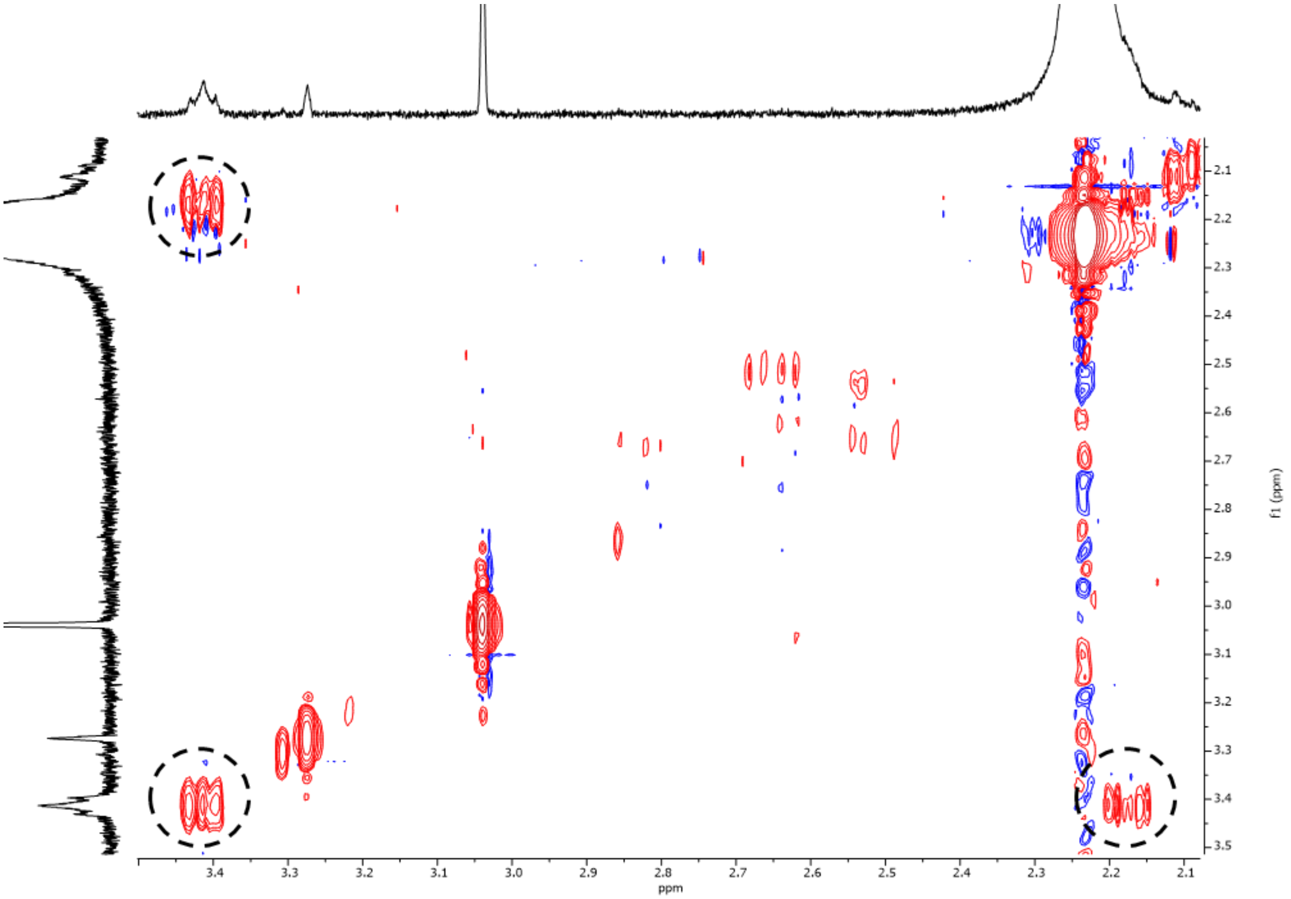

Figure S10. Relevant portion of 2D COSY NMR spectrum. Contact between signals at 3.41 ppm and 2.16 ppm is highlighted by black-dashed circles.

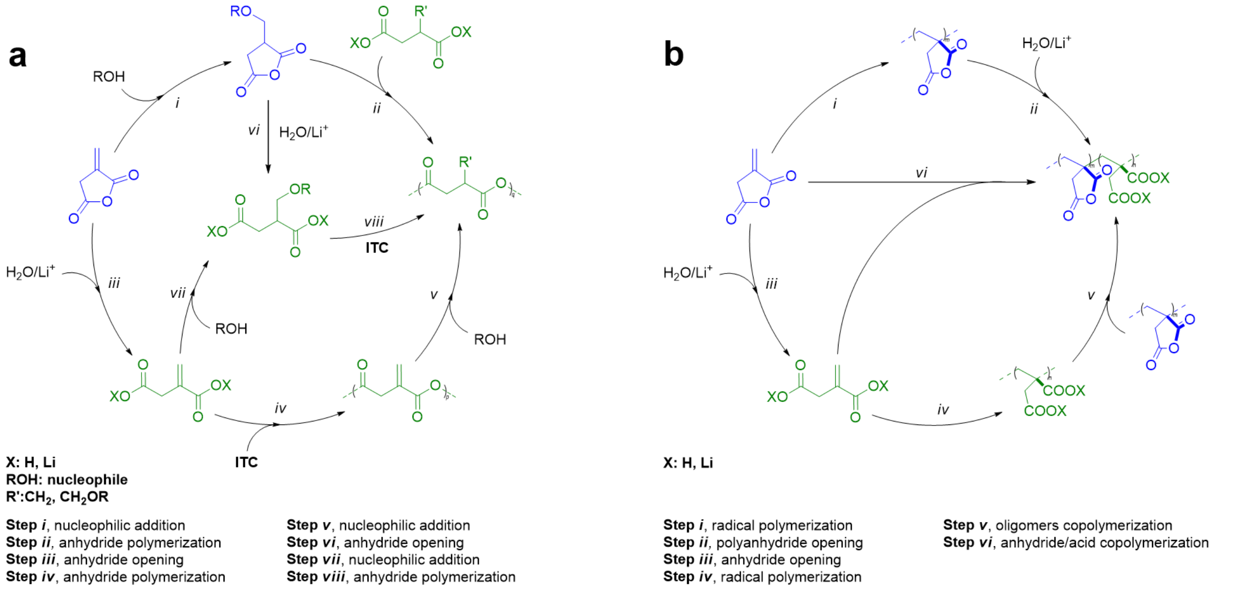

Figure S11. Proposed SEI-film formation pathways using itaconic anhydride leading to (a) linear and (b) branched polymeric structures.[8]

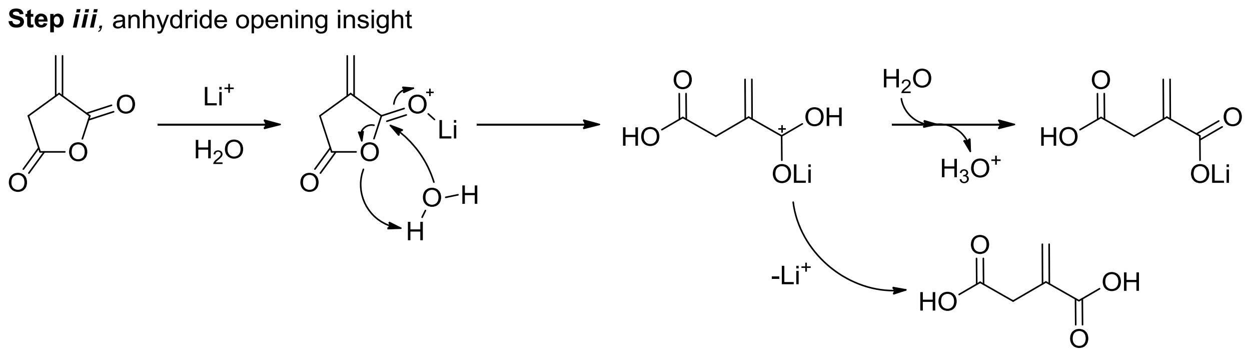

Figure S12. Anhydride ring opening pathway.

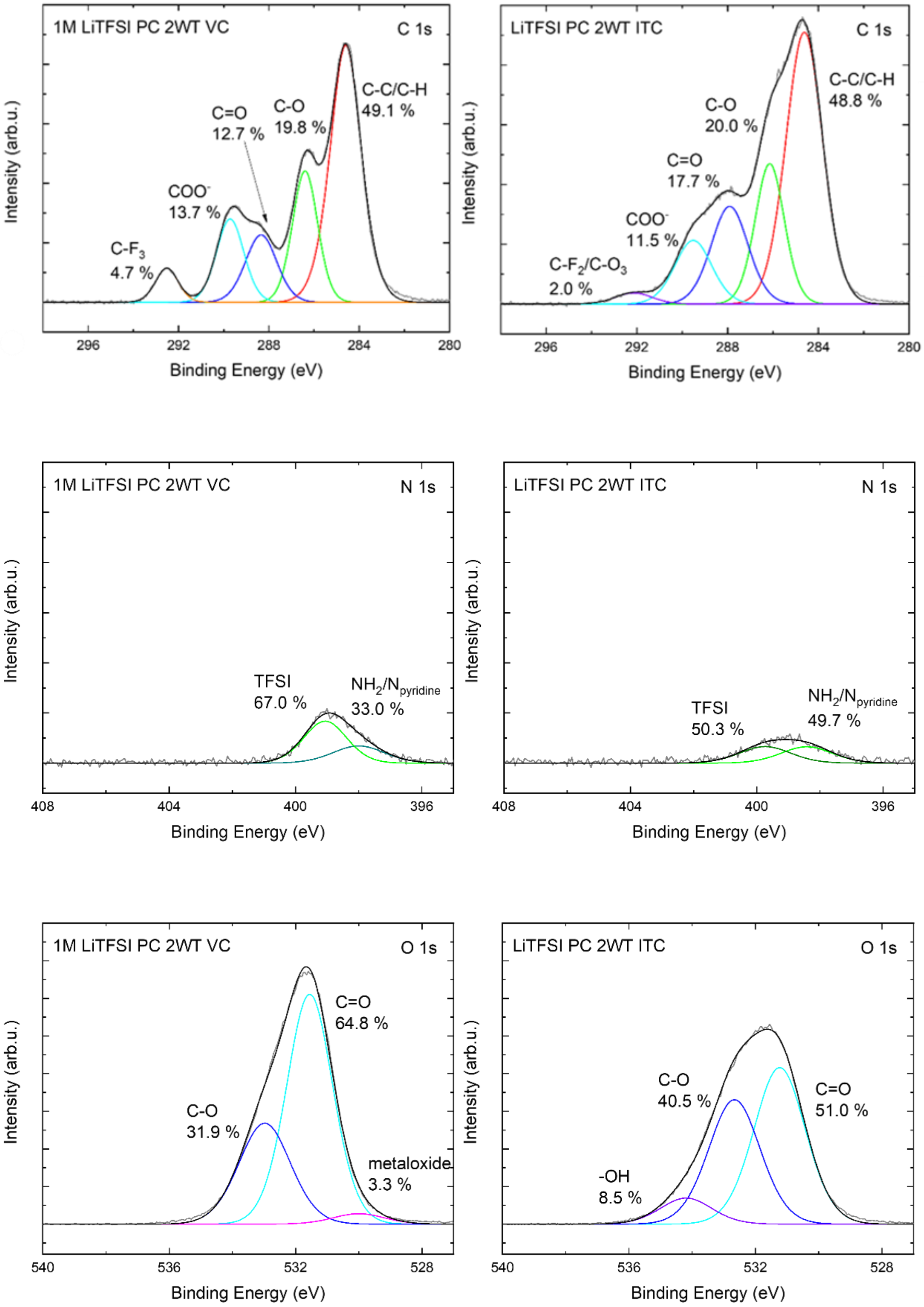
1M LiTFSI PC 2WT VC
C 1s
C-C/C-H
49.1 %
C-O
19.8 %
C=O
12.7 %
COO$^-$
13.7 %
C-F$_3$
4.7 %
Intensity (arb.u.)
Binding Energy (eV)
296
292
288
284
280
LiTFSI PC 2WT ITC
C 1s
C-C/C-H
48.8 %
C-O
20.0 %
C=O
17.7 %
COO$^-$
11.5 %
C-F$_2$/C-O$_3$
2.0 %
1M LiTFSI PC 2WT VC
N 1s
TFSI
67.0 %
NH$_2$/N$_{pyridine}$
33.0 %
408
404
400
396
LiTFSI PC 2WT ITC
N 1s
TFSI
50.3 %
NH$_2$/N$_{pyridine}$
49.7 %
1M LiTFSI PC 2WT VC
O 1s
C=O
64.8 %
C-O
31.9 %
metaloxide
3.3 %
540
536
532
528
LiTFSI PC 2WT ITC
O 1s
C-O
40.5 %
C=O
51.0 %
-OH
8.5 %

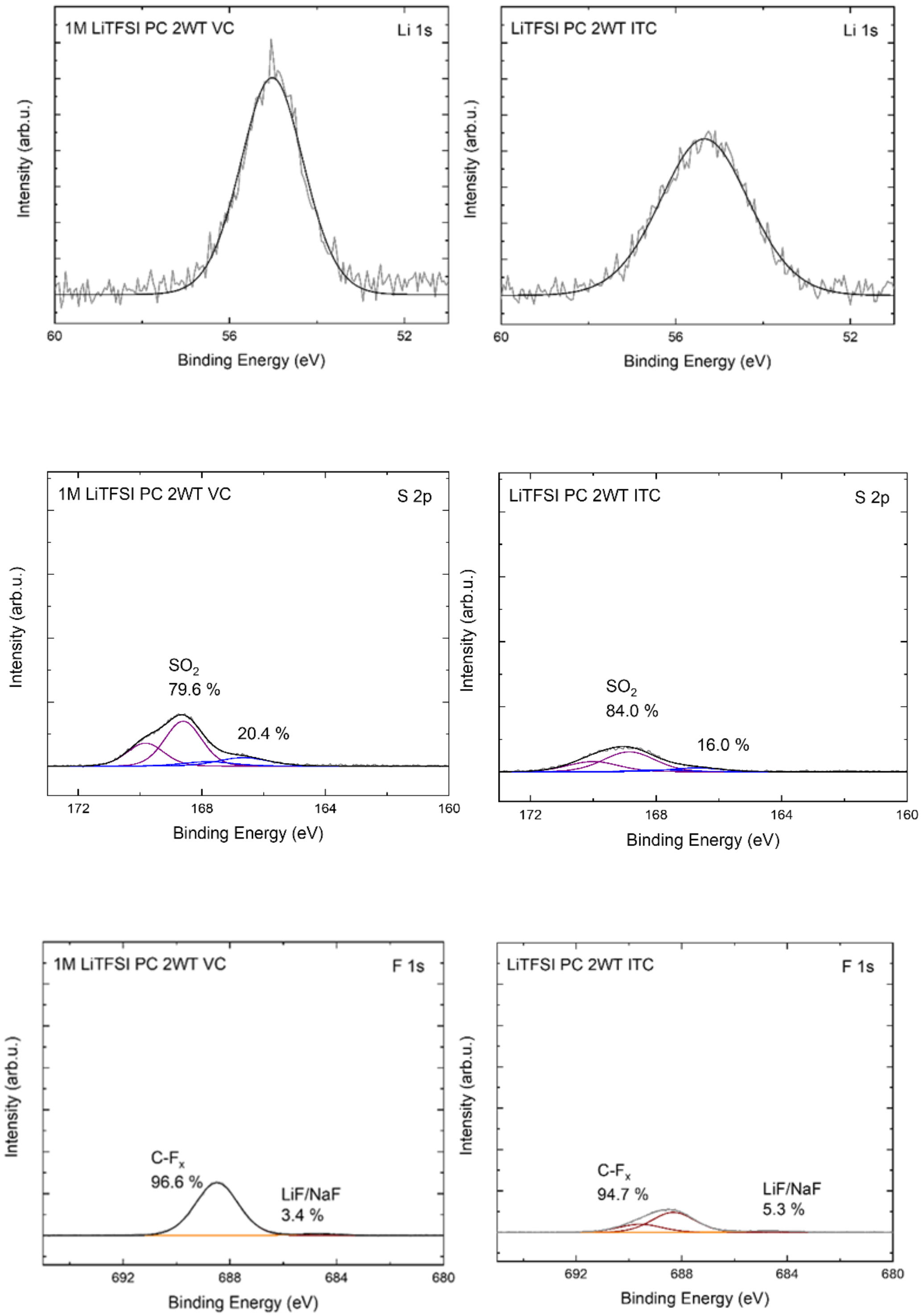

Figure S13. C 1s, N 1s, O 1s, Li 1s, S 2p and F 1s spectra of graphite electrodes cycled in 1 M LiTFSI in PC with 2 wt% VC and 2 wt% ITC.

References

[1] Thermo Scientific Chemicals. Itaconic anhydride 98%, can be found under https://www.thermofisher.com/order/catalog/product/de/de/412940250 (accessed May 13, 2025).

[2] Sigma-Aldrich. Vinylene carbonate battery grade, 99.5%, can be found under https://www.sigmaaldrich.com/DE/de/product/aldrich/809977 (accessed May 13).

[3] National Center for Biotechnology Information (2025). PubChem Compound Summary for CID 75110, Itaconic Anhydride, can be found under https://pubchem.ncbi.nlm.nih.gov/compound/Itaconic-Anhydride (accessed May 13, 2025).

[4] National Center for Biotechnology Information (2025). PubChem Compound Summary for CID 13385, Vinylene carbonate, can be found under https://pubchem.ncbi.nlm.nih.gov/compound/Vinylene-carbonate (accessed May 13, 2025).

[5] M. A. Cabañero, M. Hagen, E. Quiroga-González, *Electrochim. Acta* **2021**, *374*.

[6] T. Okuda, K. Ishimoto, H. Ohara, S. Kobayashi, *Macromolecules* **2012**, *45*, 4166-4174.

[7] a) A. Wang, S. Kadam, H. Li, S. Shi, Y. Qi, *npj Computational Materials* **2018**, *4*; b) L. Wang, A. Menakath, F. Han, Y. Wang, P. Y. Zavalij, K. J. Gaskell, O. Borodin, D. Iuga, S. P. Brown, C. Wang, K. Xu, B. W. Eichhorn, *Nat Chem* **2019**, *11*, 789-796; c) E. G. Leggesse, R. T. Lin, T. F. Teng, C. L. Chen, J. C. Jiang, *J Phys Chem A* **2013**, *117*, 7959-7969.

[8] S. Chanda, S. Ramakrishnan, *Polymer Chemistry* **2015**, *6*, 2108-2114.
